# Age of red blood cells and mortality in the critically ill

**DOI:** 10.1186/cc10142

**Published:** 2011-04-15

**Authors:** Ville Pettilä, Andrew J Westbrook, Alistair D Nichol, Michael J Bailey, Erica M Wood, Gillian Syres, Louise E Phillips, Alison Street, Craig French, Lynnette Murray, Neil Orford, John D Santamaria, Rinaldo Bellomo, David J Cooper

**Affiliations:** 1 Australian and New Zealand Intensive Care Research Centre, Department of Epidemiology and Preventive Medicine, Monash University, Commercial Road, Melbourne 3004, Victoria, Australia; 2 Department of Intensive Care and Hyperbaric Medicine, The Alfred Hospital, Commercial Road, Melbourne 3004, Victoria, Australia; 3 Australian Red Cross Blood Service, St Kilda Road, Melbourne 3004, Victoria, Australia; 4 Transfusion Outcomes Research Collaborative, Department of Epidemiology and Preventive Medicine, School of Public Health and Preventive Medicine, Monash University, Commercial Road, Melbourne 3004, Victoria, Australia; 5 Haematology Unit, The Alfred Hospital, Commercial Road, Melbourne 3004, Victoria, Australia; 6 Department of Intensive Care, Western Health, Gordon Street, Fitzroy 3011, Victoria, Australia; 7 Department of Intensive Care, The Geelong Hospital, Ryrie Street, Geelong 3220, Victoria, Australia; 8 Intensive Care Unit, St Vincent's Hospital, Victoria Parade, Fitzroy 3065, Victoria, Australia

## Abstract

**Introduction:**

In critically ill patients, it is uncertain whether exposure to older red blood cells (RBCs) may contribute to mortality. We therefore aimed to evaluate the association between the age of RBCs and outcome in a large unselected cohort of critically ill patients in Australia and New Zealand. We hypothesized that exposure to even a single unit of older RBCs may be associated with an increased risk of death.

**Methods:**

We conducted a prospective, multicenter observational study in 47 ICUs during a 5-week period between August 2008 and September 2008. We included 757 critically ill adult patients receiving at least one unit of RBCs. To test our hypothesis we compared hospital mortality according to quartiles of exposure to maximum age of RBCs without and with adjustment for possible confounding factors.

**Results:**

Compared with other quartiles (mean maximum red cell age 22.7 days; mortality 121/568 (21.3%)), patients treated with exposure to the lowest quartile of oldest RBCs (mean maximum red cell age 7.7 days; hospital mortality 25/189 (13.2%)) had an unadjusted absolute risk reduction in hospital mortality of 8.1% (95% confidence interval = 2.2 to 14.0%). After adjustment for Acute Physiology and Chronic Health Evaluation III score, other blood component transfusions, number of RBC transfusions, pretransfusion hemoglobin concentration, and cardiac surgery, the odds ratio for hospital mortality for patients exposed to the older three quartiles compared with the lowest quartile was 2.01 (95% confidence interval = 1.07 to 3.77).

**Conclusions:**

In critically ill patients, in Australia and New Zealand, exposure to older RBCs is independently associated with an increased risk of death.

## Introduction

Anemia is extremely common in the critically ill [1] and is associated with poor outcomes [2-5]. It is therefore not surprising that 19 to 53% of all patients admitted to adult ICUs receive at least one unit of allogeneic red blood cells (RBCs) [1,6-8].

Several publications have highlighted that the administration of RBCs and the hemoglobin trigger used for the administration of RBCs may affect patient morbidity and mortality [9-18]. More recently, the age of RBCs has been the focus of concern as a potential cause of increased morbidity and mortality [10]. A recent review summarizing data from 27 different studies in adult patients, however, concluded that it is difficult to determine whether there is a relationship between the age of transfused RBCs and mortality [19].

The mechanism responsible for the possible adverse effects of RBCs may relate to the development of storage lesions over time. During storage, in a way that increases over time, important biochemical changes occur: a reduction in 2,3-diphosphoglycerate, hypocalcemia, cell lysis, release of free hemoglobin, changes in nitric oxide levels, alterations in pH [20,21], and increases in lipids [22], complement [23] and cytokines [24]. These changes are accompanied by increased membrane fragility, which can compromise microcirculatory flow and lead to increased red cell-endothelial cell interaction and inflammatory cytokine release [20,21]. Such changes, which serve as potential explanations for more unfavorable outcomes, may be particularly disadvantageous to critically ill patients with a higher mortality risk. In this group, indirect evidence has linked the transfusion of older RBCs with adverse clinical consequences [25]. Unfortunately, all such evidence has been retrospective and/or focused on specific patient groups. The robustness of the relationship between the age of RBCs and adverse clinical outcome is thus limited both in strength and generalizability. Yet if this link exists, the public health consequences are great, given that the transfusion of RBCs is a common treatment in the critically ill. Furthermore, exposure to even a single unit of older RBCs might be associated with unfavorable outcome independent of the effect of volume of transfused RBCs and other confounding factors.

Accordingly, we hypothesized that the maximum age of RBCs to which a critically ill patient had been exposed would have an independent relationship with hospital mortality. We tested this hypothesis by conducting a prospective multicenter observational study in a heterogeneous group of medical and surgical critically ill patients.

## Materials and methods

### Study design

We performed a prospective multicenter observational study in Australian and New Zealand ICUs. All sites that were members of the Australian and New Zealand Intensive Care Society (ANZICS) Clinical Trials Group were invited to participate, and 47 centers agreed to collect data. Each center obtained local Institutional Ethics Committee approval. Informed consent was waived at all sites. Over a 5-week period (August to September 2008) all new adult patients admitted to the ICU who received RBCs were included. Patients remained in the study until hospital death or discharge.

Patient-specific data included the following: date and time of hospital and ICU admission, gender, age, Acute Physiology and Chronic Health Evaluation (APACHE) III diagnostic code and score, and pre-existing or currently active co-morbidities. Any type of blood component given within 24 hours prior to ICU admission or during the ICU stay was recorded. The date, time and patient status (alive or dead) at hospital discharge were also noted. RBC-specific data included the age of the RBC unit at the time of transfusion and the leukodepletion status. The age of the blood was determined by subtracting the date of collection from the date of transfusion. The donation number (this number is unique to each blood donation) for every unit transfused was noted: these numbers were used to gather information specific to each RBC unit from the Australian Red Cross Blood Service and the New Zealand Blood Service.

### Data management

Data were collected using case report forms, which were completed at sites and then faxed to the study coordinating centre at the ANZIC Research Centre, Monash University, Melbourne, Australia. The case report forms were subsequently scanned to a database using an optical reader. After checking the data and repeated queries to the study sites, the missing data related to RBC transfusions constituted <1%.

### Statistical methods regarding analysis of age of RBCs

#### Maximum age of RBCs

The relationship between hospital mortality and maximum age of RBCs received was determined using logistic regression. We chose the maximum age of RBCs transfused as the independent variable to be tested because we reasoned that exposure to even a single transfusion of old RBCs may have a toxic effect and contribute to increased mortality. Furthermore, we reasoned that once exposure to red cells with storage lesions occurs, it may cause irreversible damage and influence morbidity and mortality. The association, if present, may therefore not be linear in nature. First, we tested the age of RBCs as a continuous variable. Second, according to the literature [26], the maximum age of RBCs was divided into quartiles to include a sufficient number of patients in each group, with the lowest quartile representing the freshest possible RBCs.

#### Adjustment for confounding factors

From a univariate analysis, a list of biologically plausible and statistically significant confounders were identified, including severity of illness (APACHE III score), leukodepletion status, pre-ICU transfusions, cardiac surgery, other transfused blood components, and pretransfusion hemoglobin concentration preceding the first transfusion. We further adjusted for clustering of study sites. The APACHE III scores were first obtained by linkage of the study database with the ANZICS Adult Patient Database and were available for 432 study patients. Second, multiple requests for the missing APACHE III scores were sent retrospectively to the study sites, ending up with 713 surviving patients (94.2%) and 141 out of 146 patients who died (96.6%) with an APACHE III score (compared with <1% of missing values in other study data). Hospital discharge status was re-checked at the same time.

Finally, given a possible relationship between exposure to older blood and increased mortality, we sought to further explore this relationship. A series of binomial variables were created for each possible maximum age of blood (<2days, <3days, and so forth), and a cumulative graph was plotted indicating the mortality rate for each binomial cut-off point. To visually show the relationship between mortality and the maximum age of red blood, we also provided a plot of the predicted risk of death (as derived from the multivariate logistic regression model) against the maximum age of RBCs, and a locally weighted nonparametric smoother (LOWESS) was fitted to the data. LOWESS fits simple models to localized subsets of the data to build up a function that describes the deterministic part of the variation in the data, point by point.

### Statistical analysis

Statistical analysis was performed using SAS version 9.1 (SAS Institute Inc., Cary, NC, USA). Descriptive statistics were computed separately for all study variables for all study patients. Univariate analysis was performed using chi-square tests for equal proportions, Student *t*-tests for normally distributed outcomes and Wilcoxon rank-sum tests otherwise, with results reported as percentages (*n*), means (standard errors), or medians (interquartile ranges). The results from logistic regression analysis were reported as odds ratios (ORs) (95% confidence interval (CI)). Two-sided *P *= 0.05 was considered statistically significant.

Multivariate logistic regression models were constructed using both stepwise selection and backward elimination procedures with statistically significant covariates (*P *< 0.05) remaining in the model. Models included the identified list of covariates firstly using the maximum age of blood as a continuous variable and then secondly as a predetermined categorical variable in quartiles. The final model was further assessed for goodness of fit (Hosmer-Lemeshow test), points of influence (standardized differences in parameter estimates due to deleting the corresponding observation) and clinical and biological plausibility. To ensure that the relationship between the maximum age of blood and mortality did not differ for specific subgroups, interactions between the age of RBCs and all other covariates were explored.

## Results

### Patients and participating centers

A total of 47 ICUs participated in the study (Australia, *n *= 36; New Zealand, *n *= 11). All ICU types were represented: 28 tertiary ICUs, 10 metropolitan ICUs, four rural ICUs and five private ICUs.

In total, 757 patients received one or more units of RBCs. Their demographic and clinical data are shown in Table 1. According to their APACHE III diagnostic classification, 416 (55.0%) were operative patients and 341 (45.0%) were nonoperative patients. The largest diagnostic groups were cardiac surgery patients (194, 25.6%), bacterial pneumonia (36, 4.8%), septic shock or sepsis (56, 7.3%), gastrointestinal neoplasm (23, 3.0%), nonoperative gastrointestinal bleeding (21, 3.2%), trauma (50, 6.6%), and operative gastrointestinal bleeding (15, 2.0%). The number of transfusions and the age of RBCs are included in Table 1.

**Table 1:** Patient characteristics (*n *= 757) and transfusion details

	All patients	Quartile 1	Quartiles 2 to 4	***P *value**^**a**^
Age (years)	66 (54 to 76)	65 (50 to 74)	66 (54 to 76)	0.16
Male	468 (62%)	112(59%)	356 (62%)	0.40
Cardiac surgery patients	194 (26%)	51 (26%)	143 (25%)	0.62
Trauma patients	50 (7%)	6 (3%)	44 (7%)	0.03
Sepsis patients	56 (7%)	15 (7%)	41 (7%)	0.74
Received pre-ICU				
RBCs	333 (44%)	90 (47%)	243 (42%)	0.25
Platelets	130 (17%)	33 (17%)	97 (17%)	0.90
FFP	168 (22%)	48 (25%)	120 (21%)	0.22
RBCs transfused	2 (1 to 4)	2 (2 to 3)	2 (2 to 5)	<0.0001
Average age of RBCs	14 (9.5 to 21.5)	7.5 (5.7 to 9.0)	17.6 (12.9 to 24.0)	<0.0001
Maximum age of RBCs	18 (11 to 28)	8 (6 to 9)	22 (15 to 30)	<0.0001
RBCs leukodepleted	599 (79%)	149 (78%)	450 (79%)	0.91
Pretransfusion				
Hemoglobin (g/dl)	7.7 (7.2 to 8.2)	7.6 (7.1 to 8.2)	7.7 (7.2 to 8.2)	0.50
Received platelets	180 (24%)	48 (25%)	132 (23%)	0.55
Received FFP	256 (34%)	57 (30%)	199 (35%)	0.22
ICU length of stay (days)	3.9 (1.9 to 8.6)	3.5 (1.7 to 7.1)	4.2 (1.9 to 9.2)	0.02
Hospital mortality	146 (19.3%)	25 (13%)	121 (21%)	0.015

All values expressed as number (proportion) or median (interquartile range). RBC, red blood cell; FFP, fresh frozen plasma. ^a^Quartile 1 versus Quartiles 2 to 4.

### Age of RBCs and hospital mortality

The mean (median, standard error) pretransfusion hemoglobin level was 7.8 (7.7, 0.03) g/dl. The ages of the oldest RBCs and unadjusted hospital mortalities for the quartiles of the whole study population (*n *= 757), and hospital mortalities for the quartiles of those included in the multivariate analysis (*n *= 713) according to maximum RBC age, are shown in Table 2. The hospital mortality in the lowest quartile (Quartile 1) was 25/189 (13.2%) versus 121/568 (21.3%) in Quartiles 2 to 4, with a significant (*P *= 0.01) unadjusted absolute risk reduction of 8.1% (95% CI = 2.2 to 14.0%) in hospital mortality.

**Table 2:** Unadjusted mortality rates according to quartiles of maximum age of red cells

Quartile	Age of RBCs (days)	Mortality	
		
		All patients	APACHE III scored
1	7.7 (2 to 11)	25/189 (13.2%)	24/185 (13.0%)
2	13.8 (11 to 18)	41/189 (21.7%)	40/175 (22.9%)
3	22.6 (18 to 28)	36/189 (19.1%)	34/176 (19.3%)
4	34.4 (28 to 42)	44/190 (23.2%)	43/177 (24.3%)
2 to 4	22.7 (11 to 42)	121/568 (21.3%)	117/528 (22.1%)

Values expressed as median (range) or number/total (proportion). RBC, red blood cell; APACHE, Acute Physiology and Chronic Health Evaluation.

### Adjustment for confounding factors

In these 713 patients, there was no significant independent association with hospital mortality and the maximum age of RBCs as a continuous variable (univariate OR 1.02, 95% CI = 1.003 to 1.04, *P *= 0.025; multivariate OR = 1.02, 95% CI = 0.99 to 1.04, *P *= 0.15), but there was a statistically significant difference in mortality between quartiles of maximum age of RBCs at both the univariate level (*P *= 0.01) and the multivariate level (*P *= 0.03). Day 11 was the 25th percentile of the oldest RBC transfused (not the 25th percentile of all transfused RBCs). When compared with the lowest quartile (Quartile 1), exposure to the combination of three quartiles (Quartiles 2 to 4) of maximum age of RBCs was associated with an increased risk of hospital mortality (adjusted OR = 2.01, 95% CI = 1.07 to 3.77). Other variables independently associated with hospital mortality were APACHE III score, fresh frozen plasma transfusion, pretransfusion hemoglobin level, and cardiac surgery (for ORs see Table 3). The study site (clustering), leukodepletion status, number of RBC transfusions and pre-ICU transfusions (RBCs, platelets, fresh frozen plasma yes/no) did not show an independent association with hospital mortality.

**Table 3:** Univariate and multivariate logistic regression analysis in patients with APACHE III scores

Variable	Unadjusted		Multivariate	
	
	Odds ratio (95% CI)	*P *value	Odds ratio (95% CI)	*P *value
APACHE III score (one point)	1.03 (1.02 to 1.04)	<0.0001*	1.04 (1.03 to 1.05)	<0.0001*
RBC units transfused (number)	1.09 (1.05 to 1.13)	<0.0001*	1.02 (0.97 to 1.08)	0.45
Platelet transfusion (yes/no)	1.79 (1.20 to 2.67)	0.005*	1.17 (0.58 to 2.34)	0.66
FFP transfusion (yes/no)	2.10 (1.44 to 3.05)	0.0001*	1.98 (1.16 to 3.38)	0.01*
Cardiac surgery (yes/no)	0.21 (0.11 to 0.39)	<0.0001*	0.31 (0.14 to 0.71)	0.006*
Pretransfusion hemoglobin (per g/dl)	1.02 (1.00 to 1.04)	0.04*	1.06 (1.03 to 1.09)	0.0001*
Older quartiles versus freshest quartile of maximum RBC age	1.87 (1.17 to 2.99)	0.01*	2.01 (1.07 to 3.77)	0.03*
Leukodepletion	1.12 (0.71 to 1.77)	0.61	0.88 (0.34 to 2.24)	0.78
Study site		0.12		0.30

APACHE, Acute Physiology and Chronic Health Evaluation; CI, confidence interval; FFP, fresh frozen plasma; RBC, red blood cell. *Significant variable (*P *< 0.05).

**Figure 1:**
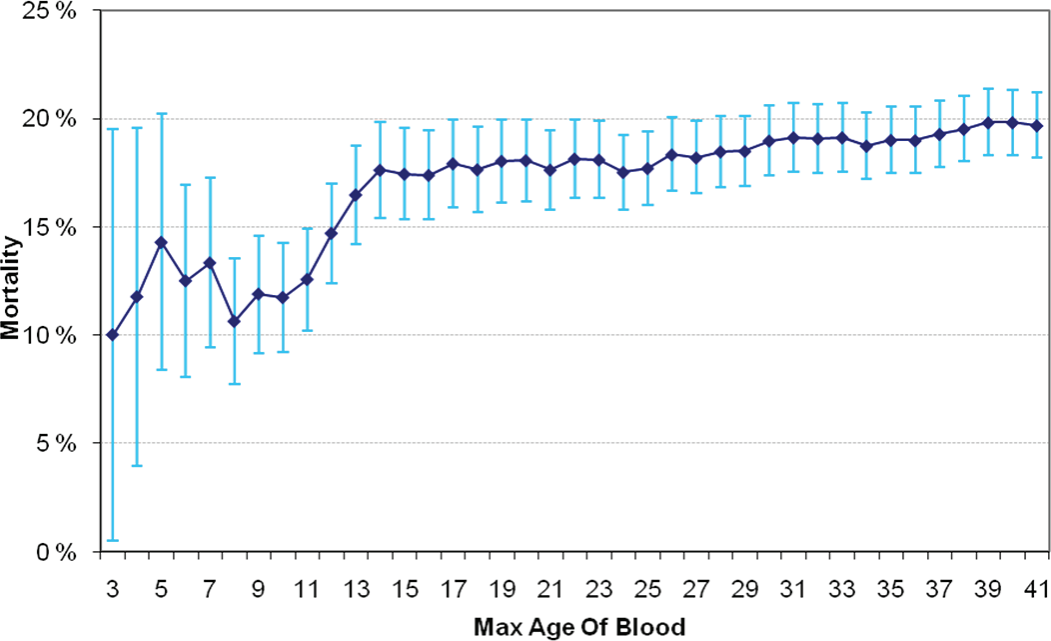
**Hospital mortality according to maximum age of red blood cells**. Hospital mortality (%, 95% confidence interval) according to the maximum age of red blood cells (RBCs) (days). Patients with the maximum age of RBCs exceeding each cut-off point are excluded.

**Figure 2:**
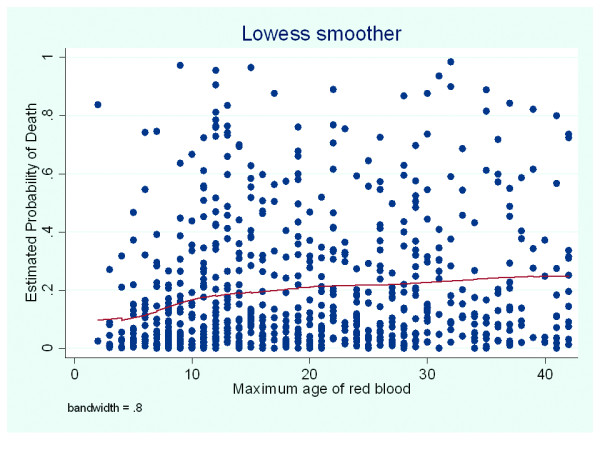
**Predicted risk of death against maximum age of red blood cells**. A locally weighted nonparametric smoother (LOWESS) for the predicted probability of death and the maximum age of red blood cells.

The area under the curve for the multivariate model was 0.86, and a Hosmer-Lemeshow *P *= 0.93 suggested the model adequately fitted the data. A graphic trend for the adjusted hospital death according to the maximum age of RBCs is presented in Figure 1 for illustration. There were no significant interactions between the maximum age of blood and all other variables in the multivariate model. In addition, the predicted risk of death against the maximum age of RBCs with LOWESS is presented in Figure 2.

## Discussion

We conducted a prospective observational study in 47 ICUs in Australia and New Zealand to assess the association between age of RBCs and outcome. In critically ill patients receiving RBCs, we found an association between exposure to older red cells and increased hospital mortality rate. This association remained after adjustment for potential confounding factors.

In this study, the mean age of all RBCs was 16.2 days and the oldest RBC unit given to each patient was 19.6 days on average. This compares with 21.2 days in the United States [1] and 16.2 days in Europe [7]. In 2007, the mean calculated age of transfused RBCs in the United States was 19.5 days, although just 7.8% of the hospitals reported such data [27]. Our results, therefore, are in agreement with the mean age of RBCs in previous studies and in other countries.

The mean pretransfusion hemoglobin values in previous studies - namely 8.6 g/dl in the United States [1] and 8.4 g/dl in Europe [7] - are in line with our mean pretransfusion hemoglobin concentration. In a previous study in Australia and New Zealand conducted in 2001 by French and colleagues the median pretransfusion hemoglobin level was 8.2 g/dl [6], compared with 7.7 g/dl in the present study. In keeping with published evidence [9], therefore, Australian and New Zealand transfusion practice appears to have moved toward a more restrictive approach during recent years.

There is no suitably powered randomized controlled trial of the effect of age of RBCs on mortality [28]. Moreover, with the exception of cardiac surgery patients, no prospective cohort study of adequate sample size has evaluated the possible association between RBC age and mortality in the critical care setting. In trauma patients, four small single-center cohort studies have suggested that exposure to older RBCs may be an independent risk factor for multiple organ dysfunction [29], increased infections [14], and increased ICU length of stay [30] and hospital length of stay [31], but none have assessed its link with mortality. Our prospective multicenter cohort study is therefore the first to assess the independent relationship between the age of RBCs and hospital mortality in a heterogeneous population of critically ill patients. Nonetheless, our findings must be seen in light of three recent large retrospective studies in cardiac surgery patients [10], in trauma patients [32], and in a registry of hospitalized patients [33].

In a study of 6,002 cardiac surgery patients, Koch and colleagues found that patients given older RBCs had an increase in unadjusted mortality, prolonged ventilation and increased sepsis, and that the transfusion of older RBCs was independently associated with an increased risk-adjusted rate of a composite of serious adverse events [10]. Although the findings of the above study are both important and provocative and the sample size was large, several features of its design made confirmatory studies desirable. First, the study was retrospective with all the inherent shortcomings of such a design. Second, the study focused only on cardiac surgery patients. Third, the study excluded more than 28% of patients because those patients received both fresh and older RBCs. Fourth, the study separated patients into two groups only according to the age of RBCs using an arbitrary 14-day cut-off point. Finally, the study did not adjust for baseline differences, age or number of units transfused before ICU treatment, and combined intraoperative and postoperative RBC transfusions [26,34].

Recently, Weinberg and colleagues demonstrated a higher mortality among trauma patients who received at least three RBC units [32]. In concordance, the largest registry study in recipients of RBC transfusion from 1995 to 2002 by Edgren and colleagues suggested that RBCs older than 30 days were associated with an increased risk of death in a 2-year follow-up [33].

Whilst impressive in sample size the retrospective registry studies have been performed mostly outside the critical care setting with a lower expected mortality rate and, thus, a lesser ability to detect relative reduction in risk. Therefore, because of the limitations of the previous studies and the public health importance of this issue, we considered it desirable to conduct a prospective, multicenter study to confirm or refute these findings in a broader population of critically ill patients.

We initially found a difference in unadjusted mortality rates according to the maximum age of red cells to which a patient had been exposed: the quartiles with older red cells were associated with a clear increase in mortality when compared with the lowest RBC quartile. However, we reasoned that this difference required correction for illness severity. Accordingly, to more rigorously test the validity of our findings, we performed multivariate analysis in these patients. We adjusted for both APACHE III score, number of transfusions, pre-ICU transfusions, fresh frozen plasma and platelet transfusions, leukodepletion status, pretransfusion hemoglobin concentration, clustering of study sites, and cardiac surgery, and we used hospital mortality as the dependent variable and found a significant and independent association between the maximum age of red cells to which a patient had been exposed and mortality. Our findings indicating an association between exposure to older RBCs and increased mortality are in broad agreement with the results of the three large retrospective studies [10,32,33], and with a *post hoc *analysis of a randomized controlled trial in critically ill children by Gauvin and colleagues [35]. The association between higher transfusion hemoglobin and higher mortality may reflect physician attempts to compensate for more severe underlying disease (for example, chronic pulmonary or cardiovascular or cerebrovascular disease) or ongoing bleeding.

The present study has several strengths. The investigation was a prospective, multicenter study and included a heterogeneous group of critically ill patients, increasing its generalizability. In addition, the study included multivariate adjustment for baseline characteristics, illness severity and relevant variables using in-hospital mortality as an endpoint.

The study also, however, has some significant limitations. This study was not a randomized trial, thus any association detected by multivariate regression analysis does not imply causation. For example, there may have been factors that influenced this association of which we are not aware and were unable to correct for (for example, use of vasopressors, PaO_2_/FiO_2 _ratios, use of antibiotics). Treating clinicians were not blinded to the age of RBCs. We have no reason to believe, however, that clinician behavior was influenced by or itself influenced the age of transfused RBCs, a variable outside their control. We did not obtain data on red cell transfusion outside the ICU. We did not follow-up patients after hospital discharge to establish their 90-day survival; such follow-up might have affected our findings. The study comprised only Australian and New Zealand ICUs and its findings may not apply to other healthcare systems. The transfusion practice and the mean age of transfused red cells, however, appear similar to those reported in studies from Europe and North America. The maximum age of red cells was not significantly associated with hospital mortality when evaluated as a continuous variable, but had a significant association when evaluated using quartiles, which can be explained by the nonlinear association demonstrated in Figure 1. In addition, our exploratory *post hoc *analysis suggests that a linear relationship between the age of blood and mortality may exist for RCBs with a lower maximum age (<15 days old), but that, beyond approximately 15 days, the deleterious effects may be less. The missing linear relationship across the whole range of RBC's age is biologically plausible given the possibility of a maximum level of deleterious changes in RBCs over time. It is also conceivable that the use of a maximum value may not readily lend itself to a linear relationship. Finally, the unadjusted difference in hospital mortality was high, raising some uncertainty about biological plausibility. In response, we adjusted for all relevant available confounding factors, expecting the difference to lose statistical significance; it did not.

## Conclusions

We conclude that, in critically ill patients in Australia and New Zealand who received RBCs, exposure to older RBCs is independently associated with increased hospital mortality compared with exposure to only the RBCs with the lowest quartile of maximum age. This observation now requires further investigation in other geographical and healthcare jurisdictions, and, if confirmed, justifies prospective randomized interventional studies to confirm or refute its impact on patient outcome.

## Key messages

• Critically ill patients treated with RBCs of the lowest quartile of maximum age had an unadjusted absolute risk reduction in hospital mortality of 8.1% compared with the other quartiles.

• This relationship remained significant after adjustment for confounding factors (OR = 2.01, 95% CI = 1.07 to 3.77).

• An adequately-sized multicentre randomized controlled trial focusing on the effect of age of RBCs and mortality in the critically ill is justified.

## Abbreviations

ANZICS: Australian and New Zealand Intensive Care Society; APACHE: Acute Physiology and Chronic Health Evaluation; CI: confidence interval; FiO_2_: fraction of inspired oxygen; ICU: intensive care unit; LOWESS: locally weighted nonparametric smoother; OR: odds ratio; PaO_2_: partial pressure of oxygen in arterial blood; RBC: red blood cell.

## Competing interests

EMW is a full-time employee of the Australian Red Cross Blood Service. The other authors declare that they have no competing interests.

## Authors' contributions

AJW, ADN, MJB, DJC, GS, EMW, AS, CF and RB were involved in the study design. GS, LM, AJW, ADN, JDS, NO and VP collected the data. MJB performed the statistical analysis. VP and RB drafted the first manuscript. All authors participated in drafting and revision of the manuscript. All authors were involved in data acquisition, and read and approved the final manuscript.
